# Efficacy of black cohosh (*Cimicifuga racemosa* L.) in treating early symptoms of menopause: a randomized clinical trial

**DOI:** 10.1186/1749-8546-8-20

**Published:** 2013-11-01

**Authors:** Sakineh Mohammad-Alizadeh-Charandabi, Mahnaz Shahnazi, Jila Nahaee, Somaei Bayatipayan

**Affiliations:** 1 Midwifery Department, Tabriz University of Medical Sciences, Tabriz, Iran

## Abstract

**Background:**

This study aims to evaluate the efficacy of Black cohosh (*Cimicifuga racemosa* L.) in treating early menopausal symptoms.

**Methods:**

This randomized, double-blind, placebo-controlled clinical trial was conducted on 84 early post-menopausal participants with Greene climacteric scale (GCS) scores of 15 to 42, who were referred to two public health care centers in Tehran, Iran, in 2011–2012. The participants were randomly allocated into treatment (6.5 mg of dried extract of Black cohosh roots daily) and control (placebo) groups with a ratio of 1:1. The participants took one tablet per day for 8 weeks. The GCS scores were recorded at baseline, and after 4 and 8 weeks of treatment. Data analysis was carried out using a general linear model with repeated measures with SPSS software. The level of significance was set at *P* < 0.05.

**Results:**

There was no loss to follow-up during the 8 weeks of treatment. The GCS total score (primary outcome) in the treatment group was significantly lower than that in the control group at both week 4 [adjusted mean difference: -7.8 (95% confidence interval: -11.1 to -4.4)] and week 8 [-12.9 (-16.2 to -9.3)]. The treatment group showed significantly more improvement than the control group in all GCS subscale scores (vasomotor, psychiatric, physical, and sexual symptoms; secondary outcomes). The differences between the treatment and control groups at week 8 were significantly higher (*P* < 0.001) than those at week 4 in terms of the total scores and the vasomotor and psychiatric subscale scores. No side effects were reported.

**Conclusions:**

Black cohosh reduced the GCS total score and all GCS subscale scores (vasomotor, psychiatric, physical, and sexual symptoms) during 4 and 8 weeks of treatment.

**Clinical trial registration:**

This study was approved (Code 9061) by the Ethics Committee of Tabriz University of Medical Sciences and registered at the Iranian Registry of Clinical Trials with IRCT201107186709N4 on 15 January 2012.

## Background

Menopause is the final menstrual period, and occurs at an average age of 51 years [1,2]. The number of peri-menopausal women is increasing in the world [1].

Menopause happens because of loss of ovarian activity, and is associated with a number of early and late symptoms. Early symptoms include hot flashes, insomnia, sweating, anxiety, palpitations, headaches, poor concentration, and loss of libido [3,4]. These symptoms usually last for 1 or 2 years after menopause, but may continue up to 10 years or more in some women [2]. The symptoms may reduce quality of life, and are independent of age and other sociodemographic variables [5]. In a study in Ilam, Iran, early post-menopausal women suffered vasomotor symptoms (55%), vaginal dryness and decreased libido (43%), and mild anxiety and depression (54%) [6].

Hormone replacement therapy is the standard treatment for early symptoms in post-menopausal women [7], but increases the risks of stroke, heart diseases, and breast cancer in older women [8,9]. Some studies have shown that the number of post-menopausal women using hormone replacement therapy is low [10] and that the effects of hormone replacement therapy in reducing menopausal symptoms are lower than expected [11,12]. For these reasons, there has been a tendency toward use of alternative therapies to relieve menopausal symptoms [13,14].

Black cohosh (*Cimicifuga racemosa* L.) is an herb proposed for treatment of menopausal symptoms [15]. *Cimicifuga* roots and rhizomes contain triterpene glycosides (*e.g.*, actein, 27-deoxyactein, and cimifugaside) and alkaloids (*e.g.*, cytisine and N-methyl cytisine), derivatives of phenylpropane (*e.g.*, ferulic acids and isoferulic acids), and cimicifugine (25–50% of the root components) [15]. Although the exact mechanisms underlying the effects of Black cohosh have not been determined, its medical effects are related to triterpene glycosides [16]. It functions in a serotogenic manner rather than an estrogenic manner [17-19], binds to estrogen receptors, and selectively suppresses luteinizing hormone secretion with no effect on follicle-stimulating hormone [20].

The effects of Black cohosh on early menopausal symptoms have been inconsistent [21-25]. A systematic review [26] conducted on six randomized controlled trials (RCTs) with a total of 1163 peri- and post-menopausal women concluded that the efficacy of Black cohosh in reducing the symptoms was currently not supported by fully conclusive evidence, and that further investigations were desirable. Another review [27] of 16 studies that addressed the weaknesses of previous studies and their heterogeneity found insufficient evidence to support the efficacy of Black cohosh on early menopausal symptoms.

Black cohosh is well tolerated and is generally safe [15,26,28,29]. However, some insignificant side effects, such as nausea, vomiting, headaches, and dizziness were reported [15,26]. We found no data for toxic doses of Black cohosh. No serious side-event was reported even in a study [24] using a higher dose of Black cohosh that included 128 mg/day standardized to 7.27 mg of triterpene glycosides (ethanolic extract of Black cohosh).

The Greene climacteric scale (GCS), which is used to assess the symptoms of menopause, is a comprehensive validated tool consisting of 21 questions that women use to rate how much they are bothered by the symptoms [30].

The inconclusive evidence on the efficacy of Black cohosh has resulted in inconsistent practices by clinicians regarding the prescription of Black cohosh in the study area, leading to confusion for some patients on their decision about its use. To provide more evidence in this area, this study aims to evaluate the efficacy of Black cohosh on early menopausal symptoms using an RCT.

## Methods

### Design

This study was a randomized, double-blind, placebo-controlled trial. A total of 84 menopausal women were randomly allocated into treatment and control groups based on a block randomization with block sizes of 4 and 6 and an allocation ratio of 1:1. The allocation sequence was identified by computerized random numbers. Sequentially numbered sealed envelopes of the same shape and size containing Black cohosh or placebo tablets were used to conceal the allocation and to maintain the blinding. Every envelope contained 56 pills of Black cohosh or placebo. The participants were instructed to take one tablet per day after dinner for 8 weeks.

The envelopes were prepared by a person who was not involved in the recruitment, data collection, or data analysis. Therefore, the researchers and participants were unaware of the kind of tablets given to each participant (blinding).

The Black cohosh tablets, which were bought from a pharmacy, were in the form of coated tablets. Each tablet contained 6.5 mg of dried extract of Black cohosh root, representing 0.12–0.18 mg of 27-deoxyactein. The tablets, named Cimifugol, are commercially distributed by Goldaroo Drug Company in Iran [31]. The placebo tablets were produced in the School of Pharmacy of Tabriz University of Medical Sciences. These tablets were the same as the Black cohosh tablets in shape, color, and size, and were thus indistinguishable from the Black cohosh tablets.

### Setting and participants

Women who met the following criteria were eligible for recruitment: (1) age of 45–60 years; (2) no menstrual cycle in the last 12 months; and (3) GCS score of 15–42. The exclusion criteria included: (1) high blood pressure (more than 140/90 mmHg); (2) history of breast cancer, uterine cancer, abnormal vaginal bleeding, liver disease, depression, hypertension, or hyperthyroidism; (3) use of steroids or herbal medications for treatment of menopausal symptoms or psychiatric drugs within the last 2 months; (4) sensitivity to spices or essences; (5) smoking; and (6) alcohol consumption.

The subjects were selected from women referred to two public health care centers affiliated to Beheshti University of Medical Sciences, Tehran, Iran. These centers had the highest numbers of clients for family planning services among the centers affiliated to the university. All participants had household records at the centers for receipt of primary health care, including family planning services, in previous years and had reached the age of menopause. To contact the participants, we found their phone numbers from the records. Some of the eligibility criteria were checked over the phone. The potentially eligible subjects were invited to attend the centers. At the centers, the women were further informed about the study, the eligibility criteria were checked precisely, and informed consent forms were signed by the eligible women.

The research protocol were approved (Code 9061) by the Ethics Committee of Tabriz University of Medical Sciences and registered at the Iranian Registry of Clinical Trials with IRCT201107186709N4 on 15 January 2012 (http://www.irct.ir/searchresult.php?id=6709&number=4).

The sample size was calculated using STATA software version 9.2 (Statasoft Inc., USA) based on a study by Hakimi *et al.*[32]. Considering 28.1 as the mean and 1.98 as the standard deviation for the GCS score in the control group, a reduction in the GCS total score of at least 15% by the treatment, a one-sided significance level of 0.05, a power of 0.80, and a 10% probable drop in the sample, the required sample size was calculated to be 42 participants per group.

### Outcomes

The primary outcome was the GCS total score assessed at weeks 4 and 8 of follow-up and the secondary outcomes were the GCS subscale scores (vasomotor, physical, psychiatric, and sexual symptoms) at the follow-ups as well as also possible side effects.

### Data collection

Data were collected at baseline prior to the treatment, and also at weeks 4 and 8 after commencement of the treatment using a questionnaire. The questionnaire included demographic and GCS statements [30], which were completed in face-to-face interviews by the participants at the visits. The participants were also given a form, and were requested to mark their taking of the pills and the occurrence of any side effects (*e.g.*, headaches, dizziness, nausea, and vomiting) on the form every day and return it to the researcher at the next visit. Furthermore, we checked the treatment compliance and possible side effects over the phone once a week.

The GCS scale consisted of 21 statements. Eleven statements pertained to psychiatric symptoms, and included two parts, anxiety and depression. Seven statements assessed physical aspects and two assessed vasomotor symptoms. The final statement considered sexual desire disorder. The severity of the symptoms was scored as zero (no symptoms), one (mild), two (moderate), and three (severe) based on self-reporting [30]. To determine the reliability, a test-retest was used (r = 0.96).

### Statistical analysis

The normality of the quantitative variables for each of the groups was confirmed using the Kolmogorov-Smirnov test. An independent *t*-test was used for comparison of the baseline scores, and a general linear model with repeated measures was used for comparison of the follow-up scores adjusted for the baseline values, time, and interaction between time and group. The Greenhouse-Geisser test was used to determine the interaction effect. A partial Eta^2^ value was recorded for the effect size of the treatment and the interaction. All data analyses were carried out using SPSS for Windows 13.0 (SPSS Inc., USA). Values of *P* < 0.05 were considered to indicate statistical significance.

## Results

There was no loss to follow-up and all participants continued the study to the end. Both groups were similar in terms of the personal and societal characteristics (Table 1). The average age in both groups was approximately 51 years. The majority of patients in both groups had high school education or above, and were married housewives. Overall, 17% of women in the treatment group and 24% in the control group did no exercise at all.

**Table 1:** Demographic characteristics of the study participants in the treatment (Black cohosh) and control (placebo) groups

**Variables**	**Treatment**	**Control**	**Statistical results**
	**(n = 42)**	**(n = 42)**	
	**Mean ± SD**	**Mean ± SD**	
Age (years)	51.4 ± 4	51.7 ± 4.2	t = 0.78, df = 82, *P* = 0.43
Length of menopause (months)	35.1 ± 20.6	31.4 ± 21.3	t = 0.82, df = 82, *P* = 0.41
Systolic blood pressure (mmHg)	115.7 ± 13.5	115.2 ± 8.6	t = 0.19, df = 82, *P* = 0.84
Diastolic blood pressure (mmHg)	79.2 ± 6.7	77.3 ± 6.6	t = 1.30, df = 82, *P* = 0.19
Body mass index (kg/m^2^)	27.6 ± 3.6	26.3 ± 2.9	t = 1.83, df = 82, *P* = 0.07
	**n (%)**	**n (%)**	
**Education**			
Elementary	9 (21)	6 (14)	χ^2^ = 4.49
Secondary	4 (9)	11 (26)	df = 3
High school	20 (47)	19 (45)	*P* = 0.21
Academic	9 (21)	6 (14)	
**Employment**			
Housewife	33 (78)	33 (78)	χ^2^ = 0.22
Employed	4 (9)	5 (11)	df = 2
Retired	5 (11)	4 (9)	*P* = 0.89
**Income adequacy**			
Yes	15 (35)	14 (33)	χ^2^ = 0.17
To some extent	24 (57)	24 (57)	df = 2
No	3 (7)	4 (9)	*P* = 0.91
**Exercise**			
Never	7 (17)	10 (24)	χ^2^ = 0.71
Sometimes	15 (36)	13 (31)	df = 3
Often	13 (31)	12 (28)	*P* = 0.87
Always	7 (17)	7 (17)	
**Marital status**			
Married	40 (95)	36 (86)	χ^2^ = 3.54
Single	1 (2)	2 (4)	df = 3
Divorced	1 (2)	1 (2)	*P* = 0.31
Widowed	0 (0)	3 (3)	

At baseline, the mean ± standard deviation of the GCS total score was 33.8 ± 6.4 in the treatment group and 31.1 ± 6.4 in the control group, with no significant difference. In addition, the groups had similar scores for all GCS subscales (Table 2).

**Table 2:** Comparisons of the treatment (Black cohosh) and control (placebo) groups in terms of the Greene climacteric scale total and subscale scores at baseline and two follow-up time-points

**Outcome variables**	**Treatment (n = 42) Mean ± SD**	**Control (n = 42) Mean ± SD**	**Comparison of the groups**		
			**Adjusted MD (95%CI)**	***P***	**Partial Eta**^**2**^
**Total score (0–63)**					
Baseline	33.8 ± 6.4	31.1 ± 6.3	2.7 (-0.1 to 5.4)	0.059	
4 weeks	16.7 ± 7.7	22.9 ± 9.2	-7.8 (-11.1 to -4.4)	< 0.001	0.21
8 weeks	8.0 ± 5.5	19.5 ± 11.0	-12.9 (-16.2 to -9.3)	< 0.001	0.39
**Vasomotor symptoms (0–6)**					
Baseline	4.0 ± 1.7	3.5 ± 1.6	0.5 (-2.9 to 1.2)	0.22	
4 weeks	1.8 ± 2.0	2.6 ± 1.7	-1.1 (-0.4 to -1.8)	0.002	0.11
8 weeks	0.7 ± 1.6	2.4 ± 1.8	-1.9 (-1.2 to -2.6)	< 0.001	0.26
**Psychiatric symptoms (0–33)**					
Baseline	18.8 ± 4.8	18.8 ± 4.3	0.1 (-2.1 to 1.9)	0.92	
4 weeks	10.8 ± 5.0	13.3 ± 6.4	-2.6 (-0.3 to -4.9)	< 0.001	0.16
8 weeks	4.2 ± 3.5	11.4 ± 7.4	-7.3 (-9.6 to -5.0)	< 0.001	0.31
**Physical symptoms (0–21)**					
Baseline	7.7 ± 3.5	6.6 ± 2.7	1.1 (-0.3 to 2,5)	0.10	
4 weeks	3.1 ± 2.3	4.7 ± 2.8	-2.0 (-3.1 to -1.0)	< 0.001	0.06
8 weeks	1.4 ± 1.5	3.7 ± 2.4	-2.5 (-3.3 to -1.7)	< 0.001	0.33
**Loss of interest in sex (0–3)**					
Baseline	2.6 ± 0.8	2.4 ± 1.4	0.2 (-0.7 to 0.3)	0.47	
4 weeks	1.8 ± 1.0	1.9 ± 1.0	-0.37 (-0.72 to -0.03)	0.03	0.06
8 weeks	1.6 ± 1.1	1.9 ± 1.0	-0.52 (-0.89 to -0.15)	0.006	0.09

The GCS total score in the treatment group was significantly lower than that in the control group at both week 4 [adjusted mean difference: -7.8 (95% confidence interval: -11.1 to -4.4), *P* < 0.001] and week 8 [-12.9 (-16.2 to -9.3), *P* < 0.001]. The treatment group also showed significantly more improvement than the control group in all GCS subscale scores (vasomotor, psychiatric, physical, and sexual symptoms).

At weeks 4 and 8, there were 51% and 77% reductions in the GCS total score (relative effect) in the treatment group compared with 27% and 38% in the control group, respectively. Reductions in the vasomotor symptom score of 61% and 86% were observed in the treatment group compared with 27% and 29% in the control group. The reductions in the psychiatric symptom score were 46% and 79% in the treatment group compared with 31% and 42% in the control group. The reductions in the physical symptom score were 63% and 82% in the treatment group compared with 27% and 42% in the control group. The reduction in the loss of interest in sex score were 29% and 36% in the treatment group compared with 8% and 10% in the control group. The effect size was considerable (partial Eta^2^: > 0.3) in terms of the total, physical, and psychiatric symptoms at 8 weeks of follow-up (Table 2).

**Figure 1:**
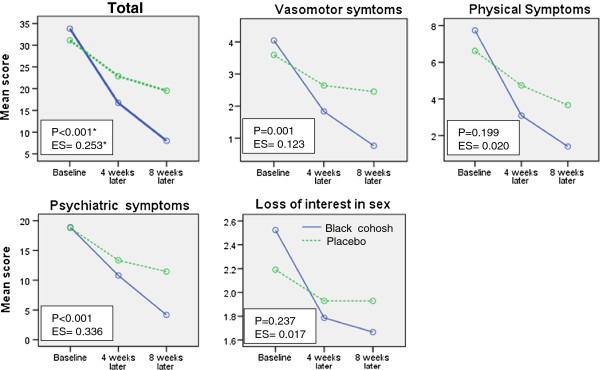
Trend in the Greene climacteric scale total score and subscale scores at the two follow-up time points (*results of the Greenhouse-Geisser test in the general linear model with repeated measures for the interaction between time and group; ES: effect size based on the partial Eta^**2**^**).**

The differences between the treatment and control groups at week 8 were significantly higher than those at week 4 in terms of the total score (*P* < 0.001) and vasomotor (*P* = 0.001) and psychiatric (*P* < 0.001) subscale scores. In the other two subscales, physical and sexual symptoms, the effects were higher at week 8, but the differences were not significant compared with week 4 (*P* = 0.199 and *P* = 0.237, respectively) (Figure 1). No side effects were reported in either of the groups.

## Discussion

Intake of Black cohosh (6.5 mg of dried extract of the roots of the plant) for 8 weeks, comprising one tablet every night after dinner, reduced the symptoms in early post-menopausal women compared with intake of placebo. The treatment efficacy increased with longer use.

The results of this study are consistent with the results of some previous studies comparing the effects of daily intake of 6.5 mg of dried rhizome extract of Black cohosh [21] or 40 mg of herbal drug Black cohosh with peri- and post-menopausal women [22,23] on reducing climacteric complaints compared with intake of placebo, and the women in the early climacteric phase benefited more than those in the late phase [22].

The effects of Black cohosh on reducing climacteric symptoms in peri- and post-menopausal women were similar to those of other effective treatment methods like conjugated estrogens [23] and tibolone [33], and even higher than those of fluoxetine [34].

The present results appear inconsistent with the results of some other studies. Geller *et al.*[24] showed that the effect of Black cohosh (64 mg/day) in reducing the number of vasomotor symptoms was not significantly different from that of placebo and was significantly lower than 0.625 mg of conjugated equine estrogens plus 2.5 mg medroxyprogesterone acetate. The difference may be related to the longer interval between the initiation of treatment and the outcome assessment, since the study outcome was assessed at 12 months after initiation of the treatment compared with 2 months in our study. Jacobson *et al.*[35] demonstrated that Black cohosh was not significantly effective in reducing most post-menopausal symptoms. However, all participants in their study had breast cancer and most (70%) were taking tamoxifen. Higher loss of follow-up (20%) may also have affected the results. Amsterdam *et al.*[25] demonstrated that Black cohosh did not significantly reduce the GCS scores in peri- and post-menopausal women. This inconsistency with our results may be related to the small sample size (only 28), relatively higher loss to follow-up (25%), or choice of Black cohosh preparation and dosage. No adverse side effects were seen in our Black cohosh users, consistent with the results of other studies [22,23,25,33,34].

The double-blinding and no loss to follow-up were important strengths of this study. The duration of the treatment in this study was relatively short (8 weeks) and we did not have any follow-up after ceasing Black cohosh use because of time and financial limitations. Although the GCS score is a subjective outcome (self-reporting), the results may not be affected by subjectivity because of the double blinding of the study. Other outcomes like urogenital and ophthalmic symptoms, follicle-stimulating hormone serum levels, and bone metabolism were not assessed, and safety was only assessed by self-reporting because of financial limitations. In addition, the effect of Black cohosh was not compared with a standard treatment, *i.e.*, hormone replacement therapy based on the results.

## Conclusions

Black cohosh reduced the GCS total score and all GCS subscale scores (vasomotor, psychiatric, physical, and sexual symptoms) during 4 and 8 weeks of treatment.

## Abbreviations

IRCT: Iranian registry of clinical trials; GCS: Greene climacteric scale; RCT: Randomized controlled trial.

## Competing interests

The authors declare that they have no competing interests.

## Authors’ contributions

SMAC and MS designed and supervised the study, and performed the statistical analyses. JN wrote the manuscript and conducted the experiments. SB assisted experimentally. All authors read and approved the final version of the manuscript.
